# Alternative Methods for the Detection of Emerging Marine Toxins: Biosensors, Biochemical Assays and Cell-Based Assays

**DOI:** 10.3390/md12125719

**Published:** 2014-11-26

**Authors:** Laia Reverté, Lucía Soliño, Olga Carnicer, Jorge Diogène, Mònica Campàs

**Affiliations:** IRTA, Carretera Poble Nou km 5.5, 43540 Sant Carles de la Ràpita, Spain; E-Mails: laia.reverte@irta.cat (L.R.); lucia.solino@irta.cat (L.S.); olga.carnicer@irta.cat (O.C.); jorge.diogene@irta.cat (J.D.)

**Keywords:** palytoxin, ciguatoxin, cyclic imines, tetrodotoxin, immunoassays, cell-based assays, hemolytic assays, receptor-binding assays, biosensors

## Abstract

The emergence of marine toxins in water and seafood may have a considerable impact on public health. Although the tendency in Europe is to consolidate, when possible, official reference methods based on instrumental analysis, the development of alternative or complementary methods providing functional or toxicological information may provide advantages in terms of risk identification, but also low cost, simplicity, ease of use and high-throughput analysis. This article gives an overview of the immunoassays, cell-based assays, receptor-binding assays and biosensors that have been developed for the screening and quantification of emerging marine toxins: palytoxins, ciguatoxins, cyclic imines and tetrodotoxins. Their advantages and limitations are discussed, as well as their possible integration in research and monitoring programs.

## 1. Introduction

Marine toxins constitute a heterogeneous group of complex chemical compounds, produced as secondary metabolites by bacteria and microalgae (e.g., dinoflagellates, diatoms, cyanophyceae). Although not fully understood, specific environmental conditions and biological cycles can modulate microalgal population dynamics and may cause the appearance of harmful algal blooms (HABs) and their toxins, which may affect the ecosystems. Among other organisms, fish or shellfish can accumulate marine toxins produced by microalgae, thus entering in the food webs and occasionally posing a threat to human consumers. Other routes of human exposure to marine toxins, apart from oral consumption of contaminated seafood, are respiration and skin contact.

The concept “emerging marine toxins” is quite subjective. It has been used by regulatory organizations such as the European Commission and the European Food Safety Authority (EFSA) [1,2,3,4,5] to focus on recently discovered marine toxins (e.g., some cyclic imines like pinnatoxins), but also those known marine toxins that appear in waters and seafood where they were previously absent (e.g., ciguatoxins, recently appearing in the eastern Atlantic, Macaronesia). “Emerging marine toxins” has also been applied to non-regulated known marine toxins, which are considered as a possible matter of concern but for which additional toxicological evidence is needed before establishing further regulations (e.g., palytoxins and brevetoxins).

The little or no toxicological information available, the structural complexity of marine toxins, and the scarcity of purified standards have hindered the development of methodologies for their detection and their subsequent regulation by the establishment of maximum permitted levels (MPLs). Mouse bioassays (MBAs) have been for years important tools to manage seafood safety and prevent risk situations for consumers. For example, the application of the MBA for the management of Paralytic Shellfish Poisoning (PSP) toxins may be, in some situations, the safest approach since other methods may not assure a good comprehension of the risk due to the complexity of this group of toxins. In addition, MBAs, among other bioassays, may be of interest to understand the toxic potential and mechanism of action of new marine toxins and may also contribute to unexplained toxicity of seafood. Nevertheless, European authorities, having specific regulations that forbid the use of laboratory animals when equivalent alternative methods exist, encourage and facilitate the development of alternative methods [6]. Moreover, as the MBAs are non-specific methods, they do not allow the quantification of individual toxins in seafood.

As for other contaminants, instrumental analysis methods have been applied to detect and quantify some marine toxins in order to fulfil regulations. For example, Amnesic Shellfish Poisoning (ASP) toxins present in seafood are quantified using high-performance liquid chromatography (HPLC-UV) [7]. In the EU, a liquid chromatography-coupled to tandem mass spectrometry (LC-MS/MS) method is currently the reference method for the quantification of lipophilic marine toxins [6], replacing completely the MBA by 2015. For PSP toxins, although the reference method in the EU is a MBA, the Lawrence HPLC method using pre-column derivatisation and fluorescence detection is also an official control method [8], but depending on the PSP toxins profile this method is not always applicable and, therefore, the correct quantification of analogues that coelute is not possible. Other HPLC methods for PSP toxins using post-column derivatisation are also available [9,10,11]. Instrumental analysis approaches have also been contemplated for emerging marine toxins. For example, cyclic imines such as gymnodimines, pinnatoxins and spirolides, can be identified and quantified by LC-MS/MS methods [12,13,14,15,16]. Ciguatoxins can also be identified by LC-MS/MS, but the application of the method in routine is difficult due to the lack of certified material, the structural complexity of the toxins group, and the low sensitivity of the current instrumentation, since these toxins are extremely powerful and may be hazardous at concentrations in fish that are difficult to detect [17,18].

Although the instrumental analysis approach is certainly a good strategy to evaluate emerging marine toxins in food, alternative methods that can provide a higher sensitivity or that may have other advantages such as shorter analysis times or lower cost, may be used as screening or quantification tools to facilitate the evaluation of multiple samples in due time and at regulatory levels. Nevertheless, for some emerging marine toxins, the combination or coupling of instrumental analysis techniques with alternative methods could be the most appropriate approach to quantify them in seafood and evaluate their risk.

In this review, we present a detailed overview of alternative or complementary methods to the MBAs and instrumental analysis techniques for the detection of some emerging marine toxins. The advantages and drawbacks of biosensors, biochemical assays and cell-based assays for the detection of palytoxin (PLTX), ciguatoxin (CTX), tetrodotoxin (TTX) and cyclic imines (CIs) are discussed.

## 2. Biosensors, Biochemical Assays and Cell-Based Assays

Biosensors, biochemical assays and cell-based assays are promising tools to overcome MBA drawbacks and to complement instrumental analysis techniques, because of their selectivity, sensitivity, ease-of-use and low cost. In this review, the term biochemical assay includes both immunoassays and receptor-binding assays (RBAs). For each toxin group, immunoassays are first described, followed by cell-based assays (CBAs) and finally RBAs (the latter sometimes being based on the same mechanism of action than CBAs but using receptors instead of whole cells). Since biosensors can be based on any of the assays, they are described at the end of each corresponding section.

Immunoassays are biochemical assays based on the immunological affinity between an antibody (Ab) and its antigen. They have been successfully used in both the screening and precise quantification of some marine toxins, because of their high selectivity and sensitivity. It is necessary to keep in mind that immunoassays are based on a structural recognition, thus they do not provide toxicological information. Moreover, Abs may have the ability to detect different toxin analogues or derivatives, if they share a structurally similar fragment, although may be to a different extent. This cross-reactivity may be advantageous or not, depending on whether the purpose is to detect the whole family of toxins (not all of them necessarily having the same toxicological potency) or just a specific one. The most common immunoassay format is the enzyme-linked immunosorbent assay (ELISA), which relies on the use of specific Abs against the target analyte, and enzymes as labels. ELISAs can be direct or indirect. The indirect format involves the use of a labelled secondary Ab against the primary Ab, and the direct one implies the labelling of the primary Ab. While the indirect assays require more steps and thus longer analysis times, the direct approach provides higher sensitivities and shorter analysis times, but the primary Ab labelling may not be straightforward. ELISAs can also be competitive or sandwich. In competitive assays the free and immobilised toxins compete for a capture Ab and, if required, a labelled secondary Ab is added to the system. In sandwich assays the target analyte is sandwiched between two Abs: a capture Ab, which is usually immobilised and recognizes the analyte of interest, and a detector Ab, which also recognizes the antigen but not at the same antigenic site. Again, labelled secondary Abs that recognize the detector Ab may be needed. Consequently, in the development of sandwich immunoassays, only large molecules with different antigenic epitopes can be targeted. Immunostrip or immunostick tests are specific types of immunoassay, which commonly use paper pads as immobilisation supports, characterized by their rapidity, portability and ease of use. Compared to other more sophisticated techniques, they are less sensitive but allow the screening of toxins *in situ* in only a few minutes by simple visual reading.

Cell-based assays (CBAs) are assays based on the toxicological effect of toxins on cells. Marine toxins usually produce a change in the physiology, the morphology or the viability of cells, which can be measured and quantified. Most CBAs require the presence of agonists or antagonist, e.g., the drugs veratridine and ouabain, in order to counteract or emphasize the action of those toxins. Veratridine is a well-known activator of the voltage-gated sodium channels (VGSCs), which binds to these channels and blocks them in an open position. Ouabain binds to the Na^+^/K^+^-ATPase pump and blocks it in a closed position, thus impeding the flux of sodium from the interior of the cells. Toxins acting on these channels and pumps, in the presence or absence of ouabain and veratridine at appropriate concentrations, will involve a specific response on cells. In this case, different toxins or analogues sharing the same mechanism of action may act on the cells to a different extent and therefore may have different toxic potency. It is necessary to differentiate those assays implementing primary cultures from those performed with established immortal cell lines. Primary cell cultures are obtained from tissues some hours or days prior to the execution of the assay. They present the advantage of reflecting, to a larger extent, the properties that the cells have in the organism, for example in regard to the presence and amount of membrane receptors where the toxins act. In that sense, these models could be more appropriate to study some mechanisms of action of the toxins and could be more sensitive than immortal cell lines. However, the use of primary cells may be more complex than immortal cell lines, as they may involve the use of laboratory animals. In addition, primary cell cultures may present a higher variability than immortal cell lines regarding their physiology and functional properties, which are related to the organism source and the cell isolation process. Despite the advantages of primary cultures in terms of mechanism of action and high sensitivities, their use in CBAs for the determination of emerging toxins has not been extensively exploited.

The hemolytic test is a specific CBA based on the lysis of red blood cells (RBCs) in the presence of compounds that alter the osmotic equilibrium. Rather than a primary culture, RBCs should be considered as tissue samples since they lack a nucleus and are terminally differentiated. RBCs contain hemoglobin in their cytoplasm. When lysis occurs after exposure of RBCs to the toxins, hemoglobin is released and its absorbance can be measured. The hemolytic test can be applied to the detection of specific marine toxins that have the ability to bind to specific ion channels located in the RBCs membranes. Like other CBAs, in order to gain specificity an antagonist is needed. Hemolytic assays may be defined taking into account the toxin mechanism of action and the RBCs origin, since variability in the response may exist depending on the source of the cells (species, population, individual). As for any toxicological assay, the time of exposure, among other parameters, should be clearly defined.

Receptor-binding assays (RBAs) are assays based on the ability of cellular receptors to bind to a specific ligand. In these assays, the competition between a labelled toxin and the toxin present in the sample for the receptor is usually carried out. Originally, ligands were labelled with radioactive moieties, but in the later years, fluorescence and chemiluminescence labels have been exploited, avoiding hazardous waste and attaining also very low limits of detection. Like in immunoassays, cross-reactivity from structurally-related toxins may exist. Since RBAs use biomolecules that have been isolated from cells, these may help to better understand the mechanism of action of toxins.

Biosensors are bioanalytical devices consisting of a biorecognition element, which specifically recognizes the analyte of interest, in intimate contact with a transducer, which converts the biorecognition event into a measurable signal. Their specificity, sensitivity, simplicity and ease of use, together with the possibility to be developed for multiplex detection and to be miniaturised for portability purposes, make the development of biosensors for marine toxins highly desirable. Most biosensors for emerging marine toxins are surface plasmon resonance (SPR) immunosensors, an optical technique that allows the detection of the toxin of interest in real time and without the need of labels. Fluorescence, fluorescence polarisation (FP), electrochemiluminescence (ECL) and electrochemical detection have also been exploited.

**Figure 1:**
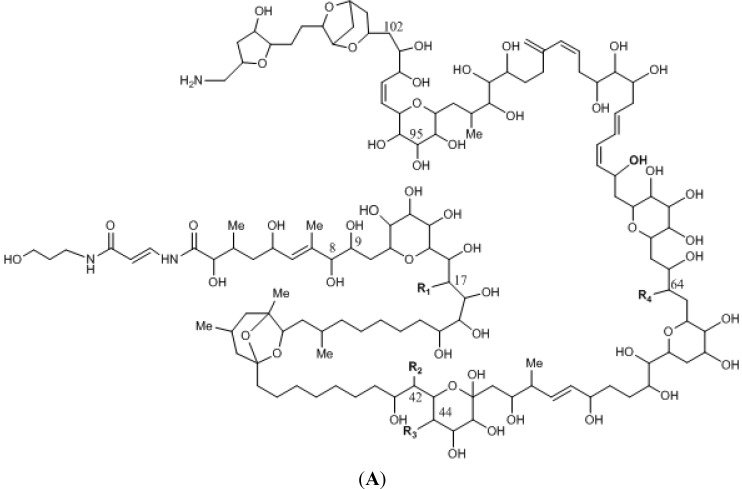

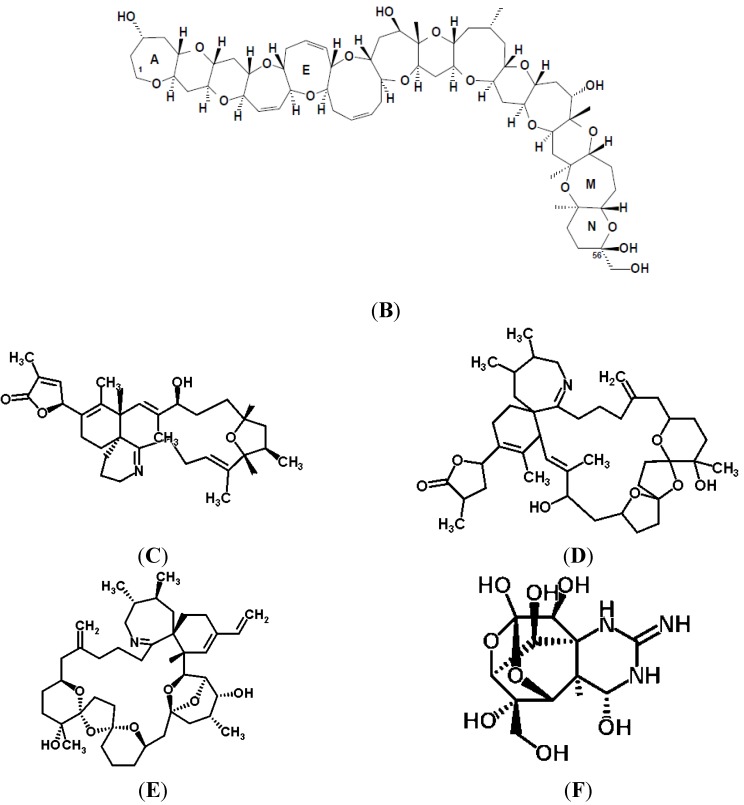
Structures of (**A**) palytoxin (PLTX); (**B**) Caribbean ciguatoxin 1 (C-CTX-1); (**C**) Gymnodimine A (GYM-A); (**D**) 13-Desmethyl spirolide C (13-DesMeC SPX) and (**E**) Pinnatoxin G (PnTX-G); and (**F**) Tetrodotoxin (TTX).

Figure 1 shows the chemical structure of some representative toxins of each toxin group, which are described in detail in the following sections.

Table 1 summarizes the emerging marine toxins included in this review, their producer organisms and vectors, the associated syndromes and effects in humans, and the geographic locations where they have been detected in food.

**Table 1:** Main features of palytoxin (PLTX), ciguatoxin (CTX), cyclic imines (CIs) and tetrodotoxin (TTX).

Toxin	Principal Derivatives	Producer Organism	Vectors	Syndromes and Effects in Humans	Endemic Areas
PLTX	Palytoxins	*Palythoa*	Fish, Molluscs, Crustaceans, Echinoderms	Clupeotoxism Rhabdomyolisis Respiratory illness Skin illness Skeletal muscle injury Digestive distress Cardiac distress	Australia, New Zealand, French Polynesia, Cook Islands, Japan, Indonesia, Micronesia, Malaysia, Vietnam, Philippines, Singapore, Hawaii, Caribbean, Mexico, Brazil, Madagascar, Reunion Island, Mediterranean coastline of Europe
	Putative PLTX Ovatoxins	*Ostreopsis* cf. *ovata*			
	Ostreotoxins	*Ostreopsis lenticularis*			
	Mascarenotoxins	*Ostreopsis mascarenensis*			
CTX	Ciguatoxins Gambiertoxins Maitotoxins	*Gambierdiscus* sp.	Fish	Gastrointestinal effects Neurological effects Cardiovascular effects	French Polynesia, Cook Islands, Hawaii, Japan, Mexico, Tokelau, North Marianna, Tuvalu, Marshall Islands, Fiji, New Caledonia, Tonga, Vanuatu, Samoa, Kiribati, Australia, Micronesia Sri Lanka, Hong Kong, Vietnam, Indonesia, Madagascar, Reunion Island, Maldive Islands, Gulf of Mexico, Guadeloupe, Florida, Virgin Islands, Puerto Rico, Brazil, Canary Islands, Madeira
CIs	Gymnodimines	*Gymnondinium selliformis*	Shellfish	Not reported	Canada, New Zealand, Japan, Denmark, Ireland, Norway, Spain, Italy, Tunisia
	Spirolids	*Alexandrium ostenfeldii*			
	Pinnatoxins	*Vulcanodinium* sp.			
	Pteriatoxins	unknown			
	Prorocentrolides	*Prorocentrum* sp.			
TTX	Tetrodotoxins	*Vibrio* sp. *Pseudomonas* sp. *Alteromonas* sp.	Pufferfish, Starfish, Blue-ring Octopus, Xanthid Crabs, Gastropods, Flatworm, Frogs, Goby, Newt Taricha	Ichthyosarcotoxism Numbness Respiratory paralysis Gastrointestinal effects	Japan, China, Taiwan, Madagascar, Australia, New Zealand, Korea, India, New Hampshire, New York, Pennsylvania, Viriginia, Chicago, California, Hawai, Bangladesh, Thailand, Norway, Meditaerranian region (Israel, Egypt and Greece), Spain (fish caught in Portugal)

### 2.1. Palytoxins

Palytoxins (PLTXs) are one of the most poisonous non-protein marine toxins. They have an acute toxicity in mice, specifically toxic by intravenous injection (lethal dose (LD50) ranging from 0.15 to 0.73 µg/kg) [19]. Despite their high toxicity in animals, few cases of human poisoning have been reported. PLTX was originally isolated from soft coral *Palythoa* sp. [20], which belongs to the family of Zoanthidae. This zoanthid was subsequently identified as *Palythoa toxica* [21]. Nearly a decade later, the structure of PLTX was described by two groups independently [22,23]. Later on, PLTX analogues named homopalytoxin, bishomopalytoxin, neopalytoxin, deoxypalytoxin and 42-hydroxypalytoxin [24] were isolated from *Palythoa tuberculosa* [25]. PLTX-group toxins are large and complex molecules with a polyhydroxylated and partially unsaturated aliphatic backbone, which contains 64 chiral centers and presents both lipophilic and hydrophilic regions [26]. The molecular weights of PLTX and its analogues differ from 2659 to 2680 Da.

Furthermore, other PLTX analogues were described in different species belonging to the benthic dinoflagellate *Ostreopsis* in tropical areas: ostreotoxins 1 and 3 in *O. Lenticularis* [27], ostreocin-D in *O. Siamensis* [28,29], and mascarenotoxins [30] in *O. mascarenensis*. In recent decades, several studies have associated *O.* cf. *ovata* seasonal blooms in the Western Mediterranean with respiratory illness in humans [31] and adverse effects in marine organisms [32] (e.g., sea urchins lost their spines and died). PLTX-like compounds have been identified in the Mediterranean strains, such as putative palytoxin (pPLTX) [33], ovatoxins-a [33], -b, -c, -d [34] and -f [35]*.* PLTXs congeners have also been identified worldwide in echinoderms, molluscs (shellfish and cephalopods), crustacean and fish [36,37].

Great efforts have been made to clearly understand the mechanism of action of PLTX. At a cellular level, PLTX blocks the Na^+^/K^+^-ATPase pump [38]. The activity of this transmembrane protein is essential for the maintenance of the cell homeostasis, which actively transports 3 Na^+^ out of the cell and 2 K^+^ in, by hydrolysing ATP under normal conditions. PLTX binds to the extracellular part of the Na^+^/K^+^-ATPase, inhibiting the active ion exchange by converting the pump into a permanently open ion channel, eventually causing cell lysis [39]. Moreover, an increase in the cytosolic Ca^2+^ caused by the action of a Na^+^-Ca^2+^ exchange pump is observed [40,41], also associated with cell death, apart from other secondary effects including cytoskeleton, cardiac and muscles contraction.

The potential routes of human exposure to PLTXs are seafood consumption and dermal and inhalation exposure to aerosols from *Ostreopsis* sp. blooms [31]. The most commonly reported symptoms of PLTX poisoning include: rhabdomyolisis, skeletal muscle injury, decrease in myocytes content in blood plasma, bitter/metallic taste, abdominal cramps, nausea, vomiting, diarrhea, paresthesia, bradycardia, renal failure, cyanosis and respiratory distress, and even death in the worst cases [42]. Additionally, clupeotoxism is a rarely occurring highly fatal form of human intoxication in tropical areas due to ingestion of clupeoid fish (some species of sardines and herrings), which was described for the first time in 1994 and attributed to the presence of pPLTX [43].

The MPLs of PLTX-group toxins in shellfish are not established. Nevertheless, in 2005 the European Union Reference Laboratory for Marine Biotoxins (EU-RLMB) set a provisional limit of 250 µg/kg of PLTX in shellfish [44]. Later on, EFSA suggested to decrease the limit to 30 µg/kg of the sum of PLTX and ostreocine-D in meat [3]. However, these are only recommendations and not regulations. Nowadays, there is no recognized official method for the determination of PLTX-group toxins.

#### 2.1.1. Immunoassays and Biosensors for Palytoxins

Contrary to TTX and CIs but similarly to CTX, the large size of PLTX is an advantage for Ab production via animal immunisation, thereby simplifying the development of immunoassays. In that sense, highly specific Abs have been raised by the immunisation of mice against PLTX conjugated to carrier proteins such as keyhole limpet hemocyanin (KLH) [45] and bovine serum albumin (BSA) [46,47,48], through the amine group of the toxin. The specificity of the produced Abs has been assessed by the development of immunoassays using both direct and indirect strategies. Two indirect competitive ELISAs have been reported so far [45,47]. While Bignami *et al.* [45] used the classical PLTX-BSA conjugate as a coating agent, Frolova *et al.* [47] took advantage of its large size and immobilised bare PLTX directly on the microtiter plate surface. The IC50 values obtained were 6.2 and 20 ng/mL, respectively. The applicability of these methods has been demonstrated by the analysis of naturally-contaminated samples of the coral *P. Tuberculosa* [45] and bacterial (*Aeromonas* sp. and *Vibrio* sp.) extracts from sea organisms including sponges, mussels and echinoderms [47]. Using the same Abs, Bignami *et al.* [45] developed two direct competitive immunoassays with the aim of reducing time and costs, using alkaline phosphatase (ALP)-labelled monoclonal antibodies (MAbs) or PLTX. Both strategies showed good sensitivity towards PLTX in spiked shellfish samples (IC50 of 3.5 and 10.1 ng/mL for MAb-ALP and PLTX-ALP, respectively), which allowed to decrease the analysis time from 4 to 2 h approximately.

Sandwich immunoassays using capture and detection Abs have also been developed for the detection of PLTX. In this direction, a direct [45] and two indirect [45,49] approaches have been reported. The direct strategy involved an ALP-labelled polyclonal antibody (PAb) and provided an IC50 of 4.8 ng/mL of PLTX. Interestingly, the same author [45] developed one of the two indirect sandwich assays, which resulted in longer analysis times but, unexpectedly, higher sensitivity (IC50 of 0.6 ng/mL). In the development of the second indirect assay [49], the affinity of the antibody towards PLTX and the analogue 42-OH-PLTX was first characterized by SPR. The affinity of the Ab seemed to depend on the method used to immobilise PLTX on the sensor chip. The ELISA assay provided an IC50 of 7.6 ng/mL and a limit of detection (LOD) of 1.1 ng/mL. Whereas Bignami’s assay [45] was applied to the analysis of the coral *P. tuberculosa*, Boscolo and co-workers [49] applied it to PLTX-spiked mussels, microalgae and seawater samples, matrices that implied only a slight shifting in the sensitivity of the assay.

Beyond the traditional method for MAb production, Garet and co-workers [50] reported the first production of highly specific recombinant Abs against PLTX using recombinant hybridoma technology. Compared to the traditional methodology, this technology presents advantages in terms of production of affinity Ab fragments without the need for animal immunisation nor antigen coupling to a carrier protein, thus reducing time and costs. In this work, the selected phage-antibody clones against PLTX were first characterized, and the most specific one was used to develop the indirect competition assay. The recombinant Ab was able to recognize free and immobilised PLTX with the best specificity ever reported up to date, even using an indirect strategy (LOD = 0.5 pg/mL). The applicability of the assay was demonstrated by the analysis of spiked samples of mussels and clams, without sample purification steps, which showed good recoveries and no or little matrix effects.

Regarding biosensors for PLTX detection, a sandwich immunoassay combined with ECL detection has been developed [48]. The immunosensor consisted of doubly amino-functionalised multi-walled carbon nanotubes (MWCNTs): CNT sidewalls were linked to MAb anti-PLTX (capture Ab) and CNT tips to the surface of an optical transparent electrode covered by an electrochemical polymer layer. The electroluminescent detection was achieved by labelling the anti-PLTX PAb (detecting Ab) with a luminescence ruthenium complex. Firstly, the affinity of the immunoconjugate (CNT-MAb) towards PLTX was evaluated by SPR. Afterwards, the electrochemiluminescent immunoassay was carried out and its applicability was demonstrated using PLTX-spiked mussels and microalgae samples. The biosensor attained an LOD of 0.07 ng/mL and a limit of quantification (LOQ) of 0.24 ng/mL, and it was also suitable for shellfish and microalgae quantification, with LODs of 0.05 and 0.06 ng/mL and LOQs of 0.22 and 0.23 ng/mL, respectively. The CNT layer enhanced the electrochemiluminescent signal, the nanocomponents favoring the electron transfer and increasing the amount of immobilised MAb compared to the system without them.

An SPR biosensor has been developed based on a direct immunoassay [51]. After characterisation of MAb kinetics and optimisation of the experimental parameters, the appropriate performance of the optical immunosensor was demonstrated. This biosensor allowed the determination of PLTX with LODs of 0.52, 2.8 and 1.4 ng/mL in buffer, grouper and clam samples, respectively.

#### 2.1.2. Cell-Based Assays for Palytoxins

CBAs for PLTX detection are based on the ability of PLTX to bind to the Na^+^/K^+^-ATPase pump, inhibiting its activity and converting it into a permanently open ion channel. As a consequence, a rapid release of K^+^ from cells is produced. In order to confer selectivity to the assay, ouabain, a specific inhibitor of the pump, is added to the system. Nevertheless, PLTX does not exactly mimic ouabain action and may not stimulate the same signaling pathways [52]. Actually, the mechanism of action is poorly understood and, at experimental level, some contradictory results have been observed.

The first CBA for PLTX was developed by Bellocci and collaborators [53] using MCF-7 cells and ouabain as an antagonist, and measuring the reduction of cytosolic lactate dehydrogenase (LDH) after cytolysis. After optimisation of the experimental parameters, the assay provided an EC50 of 530 pM of PLTX. Subsequent works [54,55,56] have used 3-(4,5-dimethylthiadol-2-yl)-2,5-diphenyltetrazolium (MTT) to measure the mitochondrial activity and quantify the cell viability, because it does not involve transferring supernatants and thus increases the reproducibility of the assay.

Comparing CBAs developed with the mouse neuroblastoma (N2a) cell strain, different findings have been reported. Ledreux and co-workers [56] observed that pre-incubation with ouabain augmented cells viability, but simultaneous addition of PLTX and ouabain increased cell death. Kebrart and collaborators [55], however, claimed increased cell death in both situations. Pawlowiez and co-workers [54] also observed amplification of the PLTX effect after pre-incubation with ouabain. Finally, Cañete and Diogène [57] reported that cell treated with ouabain and veratridine were more sensitive to PLTX than without treatment, both in N2a and neuroblastoma/glioma hybrid (NG108-15) cells. Nonetheless, and despite the differences in sensitivity, all CBAs attained EC50 values in the pM range. In fact, genetic differences between cells or between the subunits of the Na^+^/K^+^-ATPase pump, and nuances in the experimental protocol could be responsible for the encountered differences, taking into account the complexity of the mechanism of action of PLTX and its antagonist, not yet fully understood [58,59].

An interesting approach is that reported by Espiña and co-workers [60], who have developed a dynamic assay using rat hepatocytes (clone 9) and human neuroblastoma BE(2)-M17 cells. In this case, the authors used Alamar Blue, a fluorescent dye, which allowed continuous measurement of cell viability. The preventive action of ouabain on the decrease of viability caused by PLTX was more evident in human N2a cells than in hepatocytes.

Regarding the overall applicability, these CBAs have been useful to detect PLTX-like toxicity in *Ostreopsis* sp. extracts [53,54,56,60], and even in the marine cyanobacteria *Tricodhesmium* [55]. The CBA has also been applied to the determination of PLTX in spiked and naturally-contaminated seafood samples [54,56,60].

A consensus regarding PLTX extraction seems to have been achieved in recent studies focused on the *O.* cf. *ovata* strain of the Mediterranean Sea. For both microalgae cultures and seafood matrices, the most commonly used solvent is aqueous methanol, varying the proportion between 50 and 100%. For microalgae cultures, various strategies have been conducted with respect to cell collection by centrifugation or filtration, as well as for the time used to disrupt those cells by sonication [61,62,63,64,65,66].

In the early 1980s, Habermann and co-workers [67,68,69] described delayed hemolytic action of PLTX on mammal red blood cells (RBCs). Taking as a basis this effect, and a previous work with the MAb against PLTX [45], Bignami [70] developed the first hemolytic assay for PLTX using mouse blood, considered more sensitive to the PLTX effect. As previously mentioned, to verify that hemolysis is specifically due to PLTX, an antagonist should be used. Ouabain was an appropriate antagonist when using human erythrocytes; nevertheless, when using mouse blood, the anti-PLTX MAb seemed to be more effective [71]*.*

Subsequent assays have derived from Bignami’s test [70], just with slight modifications in the origin of RBCs and some experimental parameters [43,72,73]. Particularly interesting is the work performed by Riobó and co-workers [72]. Taking into account that the hemolytic effect may not be linear according to the toxin dose, and in an attempt to provide a more reliable assay, they modelled the toxicological dynamics and kinetics of hemolysis by PLTX. The model provided optimal working conditions, indicating the necessity to operate at a moderate temperature (25 °C) and to use ouabain as an antagonist.

Hemolytic assays have been applied to demonstrate the production of PTLX-like compounds by *Ostreopsis* sp. [30,37,61,62,63,64,65,66,74,75,76], as well as the presence of PLTX analogues in marine organisms such as corals, echinoderms, crustaceans and cephalopods [43,71,73,74,75,77,78,79,80,81,82,83,84] and cyanobacteria [85]. Due to the scarcity of PLTX standards availability, some authors [64,65] have decided to quantify the toxicity in relation to the saponin hemolytic activity, based on Igarashi’s method for other dinoflagellates [86].

Recently, an innovative method has been reported by Volpe *et al.* [87] to measure the hemolytic activity of PLTX. The method is based on the release of LDH from sheep erythrocytes to the medium, caused by the hemolytic activity of PLTX, and the subsequent amperometric detection of the enzyme activity on 8-screen-printed electrode strips. Ouabain was used to ensure the specificity of the assay for PLTX. The optimisation of the electrochemical detection indicated that the best approach was the use of pyruvate and NADH as enzyme substrates and phenazine methosulfate (PMS^+^). This compound reacts with the remaining NADH to produce PMSH, subsequently reacting with hexacyanoferrate (III) and producing hexacyanoferrate (II), which is oxidised on the electrode. The LOD depended on the hemolysis time, being 0.007 and 0.16 ng/mL for 24 and 4 h, respectively. Since both values are far below the proposed provisional limit, the shortest time was chosen. Compared to the spectrophotometric detection, this method is faster and allows portability.

#### 2.1.3. Receptor-Binding Assays and Biosensors for Palytoxins

Several works have developed assays for PLTX using the Na^+^/K^+^-ATPase pump. In a recent study, Na^+^/K^+^-ATPase has been labelled with a fluorescent molecule to develop a fluorescence polarisation (FP) assay [88]. The binding between PLTX and the fluorescent pump was detected by measuring the degree of polarisation of the fluorescence light. After protocol optimisation using ouabain as a model, the assay was demonstrated to be useful for PLTX quantification (LOD = 2 nM; LOQ = 10 nM), the FP decreasing proportionally to the toxin concentration. The assay was successfully applied to the analysis of mussel and dinoflagellates cultures extracts, previously cleaned up to minimize matrix interferences.

Optical biosensors, similar to the previous fluorescent assay, have been reported [89,90]. In these works, Na^+^/K^+^-ATPase pumps were immobilised on dextran-modified resonant mirrors [89] or SPR chips [90], and the PLTX binding was recorded in real time with the corresponding detector. Although the resonant mirror showed a concentration-dependent ouabain binding, no response was observed with PLTX. Since PLTX was neither able to displace bound ouabain, the authors suggested that PLTX and ouabain do not share the same binding site. The lack of response could be justified by steric impediments or the necessity of having the Na^+^/K^+^-ATPase pump inserted into the cell membrane for the PLTX binding to occur. When using SPR, the same authors immobilised the Na^+^/K^+^-ATPase via thiol coupling instead of amino coupling, with the aim of favoring PLTX binding. In this case, not only ouabain but also PLTX bound to the pump with high affinity (LOD = 3.73 pg; LOQ = 11.20 pg). The biosensor was applied to the analysis of PLTX in *Ostreopsis siamensis* cultures, demonstrating the viability of the approach.

Table 2 summarizes the biosensors, biochemical assays and cell-based assays that have been developed for the detection of PLTX.

**Table 2:** Biosensors, biochemical assays and cell-based assays for the detection of PLTX.

Assay/Biosensor	Detection Technique	Sample	Reference(s)
Immunoassay	Colorimetry	*P. tuberculosa* and spiked shellfish	[45]
		-	[46]
		*Aeromonas* sp. and *Vibrio* sp.	[47]
		Spiked mussels, microalgae and seawater	[49]
		Spiked mussels and clams	[50]
Immunosensor	ECL	Spiked mussels and microalgae	[48]
	SPR	Grouper and clams	[51]
CBA	Colorimetry	*Ostreopsis* sp.	[53,54,56]
		Spiked and naturally-contaminated seafood	[54,56]
		Cyanobacteria *trichodesmium*	[55]
		-	[57]
	Fluorescence	Spiked and naturally-contaminated seafood and *Ostreopsis* sp.	[60]
Hemolytic assay	Colorimetry	Corals (*Palythoa* sp. and *Zoanthus* sp.)	[70,77,78]
		Cyanobacteria	[81,85]
		*Ostreopsis* sp.	[30,37,61,62,63,64,65,66,74,75,76]
		Fish	[43,71,73,79,80,82]
		Sponges, soft coral, gorgonians, crustaceans	[78]
		Mouthed rock shells, sea urchin, mullet, sea-brams	[84]
		Crabs	[77]
		Clams	[37]
		Mussels	[37,75,78]
	Electrochemistry	Mussels	[87]
RBA	FP	Mussels and *Ostreopsis* sp.	[88]
Receptor-based biosensor	SPR	Ostreopsis sp.	[89,90]

### 2.2. Ciguatoxins

Ciguatoxins (CTXs) are potent polyether neurotoxins with 13–14 rings linked by ether groups into a rigid ladder-like structure [91]. They are odourless, tasteless and relatively heat-stable molecules that remain toxic after cooking and freezing, and exposure to mild acidic and basic conditions [92]. CTXs production has been associated to benthic dinoflagellates of the genus *Gambierdiscus*. The first species described was *Gambierdiscus toxicus,* originally considered as a producer of maitotoxin (MTX) and gambierols, precursors of CTXs [93,94,95]. Significant variation in toxin production occurs within the genus *Gambierdiscus* [96]. Later taxonomic studies have led to the organization of the genus into several species, reaching the conclusion that several species within this genus would produce CTXs [97], being *G. polynesiensis* a major CTX producer species [98].

CTXs can enter into the food webs through the consumption of dinoflagellates by herbivorous fish and their subsequent consumption by carnivorous fish [99]. Humans would be intoxicated by consumption of herbivorous and carnivorous fish containing CTXs. CTX precursors accumulate in fish tissue (mainly in viscera, but also in the muscle or other parts [100]) and may afterwards be metabolized into different CTX forms, which are responsible for human intoxication. Around 30 analogues from the Pacific Ocean (P-CTXs) [101,102,103,104,105,106,107,108], from the Caribbean Sea (C-CTXs) [109] and from the Indian Ocean (I-CTXs) [18,110] have been identified.

CTXs bind to VGSCs in cell membranes, blocking them in an open state, and cause membrane excitability, release of neurotransmitters, increase of intracellular calcium and blockage of voltage potassium channels [111,112,113,114,115]. This induced depolarisation of nerve cells is believed to cause some of the neurological signs associated with Ciguatera fish poisoning (CFP) [116]. CFP is the foodborne illness caused by consumption of CTX-containing fish, responsible for the highest reported incidence of human poisoning from seafood consumption worldwide [108,117]. This complex syndrome is characterized by a wide variety of symptoms such as gastrointestinal (e.g., vomiting, diarrhea, nausea), neurological (e.g., tingling, itching) and cardiovascular (e.g., hypotension, bradycardia) effects. In severe cases the symptoms may begin in 30 min after ingestion of contaminated fish, while in milder cases they may be delayed for 24 to 48 h. Fatalities may occur due to cardiorespiratory failure. At present, an estimation indicates that between 10,000 and 50,000 people are suffering around the world from this disease annually [2]. CFP is endemically found in Indo-Pacific and Caribbean areas. However, in recent years CTXs are appearing in countries not expected for their latitude, such as waters close to European and African continents, e.g., in the Canary Islands (Spain) [118] and Madeira (Portugal) [119]. This may be due, for example, to change on the distribution of toxin-producing microalgae to northern latitudes [120] or to migration of fish containing the toxins [121].

No regulatory limits exist for CTXs in fish in Europe, but the legislation requires that no fish products containing CTXs are placed on the market [4]. The United States Food and Drug Administration (US FDA) has proposed guidance levels of ≤0.1 μg/kg of C-CTX-1 equivalents and ≤0.01 μg/kg of P-CTX-1 (initially named CTX-1B, both names currently in use) equivalents [122]. In addition, since the unique existing certified standard is for P-CTX-1, toxicity equivalency factors (TEFs) for CTX congeners have been established by acute toxicity in mice (LD50) as follows: P-CTX-1 = 1, P-CTX-2 = 0.3, P-CTX-3 = 0.3, P-CTX-3C = 0.2, 2,3-dihydroxy P-CTX-3C = 0.1, 51-hydroxy P-CTX-3C = 1, P-CTX-4A = 0.1, P-CTX-4B = 0.05, C-CTX-1 = 0.1 and C-CTX-2 = 0.3. These TEFs should be applied to express individual analogues identified with quantitative detection methods as P-CTX-1 equivalents [2].

#### 2.2.1. Immunoassays and Immunosensors for Ciguatoxins

The production of specific Abs for the development of immunoassays for CTXs has been hampered by the scarcity, toxicity and chemical complexity of CTXs. The first work on this subject [123] reported the production of sheep PAbs against partially purified CTX-1B, which were subsequently radiolabelled to be used in a radioimmunoassay (RIA). Although the specificity and sensitivity were not fully optimized, this work paved the way towards the development of immunoassays for the detection of CTX in fish. These PAbs were also labelled with horseradish peroxidase (HRP) and used in a competitive ELISA [124]. The application of the assay to the analysis of CFP-related fish provided results similar to those obtained by the previous RIA and also MBA. Two years later, this ELISA was improved in terms of time and simplicity by the development of a colorimetric immunostick test [125], which was applied to the screening of fish tissue, cooked fish, soup or gravy from CFP outbreaks. The validation of this stick test was carried out with fish caught from CFP areas [126]. The test, marketed as Cigua-Check™, correlated well with the previous immunoassays. Additionally, the authors observed the presence of *Gambierdiscus toxicus* cells by microscopy in positive fish. Besides the Cigua-Check™, another immunostrip test for CTX detection, named as Ciguatect kit, was developed in 1995 by Park [127]. However, both commercial kits raised controversy regarding their performance, as disagreement with the MBA for the evaluation of CTXs was evidenced, (see comments on [128,129,130]), probably due to the use of the PAb against CTX previously developed by Hokama *et al.*, which showed high cross-reactivity with OA.

In order to overcome this limitation, Hokama and co-workers [131] produced the first MAbs against CTX, reducing the cross-reactivity towards OA to only 16%. Moreover, they developed the ELISA on oil-based paint-coated beads in order to favor the extraction and enhance the adherence of CTX. These MAbs were also exploited in a stick test format, demonstrating the ability to detect both Pacific and Caribbean congeners (P-CTX-1, P-CTX-2, P-CTX-3 and C-CTX-1) [132].

Due to the lack of pure CTXs and their high toxicity, synthetic fragments from different parts of CTX have been recently used for the development of Abs and their subsequent use in competitive [133] and sandwich [134,135,136,137,138,139,140] immunoassays. The rings of CTX are designated from A to M. Abs are usually produced against the right or the left ring fragments, being possible to rationally synthesize them with different lengths (different number of rings). The combination of Abs against different fragments has been an important advantage in the development of sandwich immunoassays, where at least two antigenic sites are needed, one Ab being used as a capture and the other one being labelled with HRP for the direct detection. Moreover, the rational design allows choosing the best strategy depending on the targeted congener. These assays have improved the LODs, being able to detect CTX at nano and even sub-nanomolar levels. Some of these sandwich immunoassays have been applied to the analysis of fish tissues from CFP outbreaks, the results correlating well with those obtained with N2a CBAs [134,135].

#### 2.2.2. Cell-Based Assays for Ciguatoxins

CBAs for CTX detection are based on the capability of CTXs to bind to the VGSCs of cells and block them, in an open state. On excitable cells, this fact results in an influx of sodium ions and cell depolarisation causing spontaneous action potential. If cells are not able to counteract this extra-sodium influx, cells die. In the assay, veratridine (which activates the VGSCs to initiate sodium channel gating) and ouabain (which inhibits the Na^+^/K^+^-ATPase pump) are added at selected concentrations maintaining 80% of cells viability. In such conditions, a toxin favoring Na^+^ influx (like CTXs) will result in cell death. Cell viability is usually quantified by means of the MTT test, which measures the mitochondrial activity. CBAs for CTX have been developed using a range of mammalian tumour cell lines, being the N2a cells the most widely used [141]. Other models, such as NG108-15 cells [142], have also been proven to be effective for CTX detection. Fairey and Ransdell [143] developed a N2a CBA to detect VGSC toxins such as CTXs, using luciferase viability as an end-point. Luciferase catalises luciferin oxidation to oxyluciferin using ATP-Mg^2+^ as a cosubstrate and in the oxidation process the chemical energy is converted into light. In the mentioned work, N2a cells were first transfected with a reporter gene containing the firefly luciferase under the transcriptional regulation of the human c-fos element. In this way, cells were provided with the capability to produce luminescence. In the assay, veratridine was used as an agonist of CTX and, after exposure to toxin, cells were lysed to release the luciferase. The luminiscence intensity was proportional to the ATP produced by viable cells. Nonetheless, the tendency is the application of N2a CBA with MTT due to its suitability, easy handling and resistance.

Brevetoxin (PbTx) and C-CTXs can co-occur in some areas such as the Caribbean and Gulf of Mexico, although they are usually considered responsible for different kinds of intoxication although they share the same mechanism of action. On one hand, blooms of *Karenia brevis* (PbTx producer) have been associated with massive fish kills as well as marine mammals, turtles and sea birds mortalities [144]; in humans, PbTx intoxication has been mostly due to shellfish consumption [145,146] although planktivorous fish can also accumulate this toxin [147]. On the other hand, CTX is responsible for CFP after finfish consumption. Latest improvements of the CBAs have been focused on discriminating CTX from PbTx. In this direction, Bottein-Dechraoui and co-workers [148] combined the CBA with a sodium channel RBA and achieved discrimination, due to the different relative potencies of the toxins and the opposite sensitivity of both assays (C-CTX-1 was always more potent than brevetoxins, but whereas for C-CTX-1 the CBA was more sensitive, for brevetoxins the RBA was more sensitive). The assay was successfully applied to the analysis of barracuda from Florida Keys, CTX being the present toxin. Interestingly, differences in the affinity of PbTx and CTX towards VGSCs from different tissues and species (rat and fish) have been shown by this group [149].

Manger and co-workers [150] have recently developed an innovative variant of the N2a CBA using flow cytometry. Unlike the traditional CBA, which uses a horizontal seeding N2a culture, this assay is performed with cells in suspension, and only veratridine is used as an agonist. Moreover, the membrane potential caused by sodium currents resulting from VGSC activation is measured at the end point of the assay using fluorescent voltage-sensitive dyes and a flow cytometer. The response of cells to CTX can be observed even without veratridine as enhancer, although an approximate 1000-fold increase in sensitivity is observed in its presence. This assay reduces the analysis time to minutes and provides more direct mechanistic data.

The applicability of the CBAs to the analysis of different matrices is also under investigation. Toxin extraction and clean-up steps are decisive to obtain a good toxin recovery and avoid matrix interferences. When analyzing flesh fish, the most commonly used protocol for toxin extraction is a modification of that for MBA [91]. All protocols are based in acetone extraction, followed by evaporation and two solvent partitions with methanol:water and hexane, and ethanol:water and diethyl ether, to remove fatty acid and polar compounds [103,151,152,153]. Additional steps with solid-phase extraction (SPE) cartridges can be also performed for further purification [154] or instead solvent partition for rapidity [155,156]. Some examples of matrices (apart from flesh or viscera fish) where CTX has been detected by CBAs are clams, urchins [54], mouse dry and fresh blood [157,158], rat plasma, urine, feces and tissues [159,160], and barracuda blood [161]. The application of different extraction and clean-up protocols appropriate for each sample type results in recovery values as high as 90%–96% (in case of mouse and rat blood [158,159,160]) and quantification limits of the order of 0.4 pg P-CTX/mL blood [159]. These examples illustrate the importance of the implementation of extraction and purification steps prior to the assay in order to gain specificity. They also demonstrate that the CBA may be appropriate to quantify CTXs in a wide range of tissues, providing LOQs under the values recommended for P-CTX-1.

When analyzing microalgae, cells are usually broken by sonication and toxins are double extracted with methanol and methanol:water (50:50) [162]. Like in flesh fish, the protocol can include additional purification steps such as solvent partition [76,98,104] and SPE clean-up [54,76]. These procedures have allowed the detection of CTXs in *Gambierdiscus* species and even cyanobacteria [54,55]. Furthermore, CBA has been successfully applied to detect CTXs dissolved in water by means of absorption resins for lipophilic toxins, without observing any resin matrix interferences [163]. In this work, additionally, fractioning of samples by SPE is proposed as a method to achieve discrimination of toxins.

CTX has also been demonstrated to have hemolytic capacity [164]. However, much work should be performed to obtain optimal LODs in the corresponding assay. Holland and co-workers [165] proposed a hemolytic assay, which applied to the analysis of different *Gambierdiscus* sp. extracts. They observed that MTX could be the principal responsible of the hemolytic activity in those microalgae samples. This response may be attributed to the different mechanism of action in Na^+^/Ca^2+^ channels among both toxins.

#### 2.2.3. Receptor-Binding Assays for Ciguatoxins

PbTXs have often been used in the development of RBAs for the detection of CTXs because of their similar mode of action (ability to bind to VGSCs) and availability. These RBAs are based on the competition of labelled and free toxin for the sodium channel receptor, different titriated PbTXs being used as tracer and/or competing free toxin for the construction of the dose-response curve. These assays have contributed to the elucidatation of the toxic potencies of CTX derivatives, although different findings have been observed. The first RBA was developed by Lewis and co-workers [103], who observed differences between C-CTX-1, C-CTX-2 and C-CTX-3 in their competition with tritiated PbTX-3. Afterwards, Poli and co-workers [166], who used tritiated PbTX-9 in the development of their assay, observed that both C-CTX-1 and PbTX-3 were approximately equipotent. This RBA was applied to the analysis of fish tissue (from a CFP outbreak in Haiti occurred in 1995), which allowed the quantification of 20 ng PbTx-3 eq/g fish tissue content, confirmed to be C-CTX-1 by LC-MS. Afterwards, as mentioned in the previous section, Dechraoui *et al.* [148] used CBA and RBA in parallel in order to differentiate C-CTX-1 from PbTX. It is important to note that, contrary to the work reported by Poli *et al.* [166], C-CTX-1 exhibited an 8-fold higher potency in the RBA than brevetoxins. The variability may be due to the receptor source and isolation process. Afterwards, Darius *et al.* [156] applied the RBA to the analysis of fish and *Gambierdiscus* populations from CFP areas in two islands of French Polynesia. In this case, a threshold toxin content was established to differentiate positive from negative fish. Interestingly, while in risky areas (where ciguatera cases are reported annually) 94% of the fish were positive by RBA, *Gambierdiscus* cells were absent. Contrary, in the area supposed to be safe, fewer fish were positive by RBA (74%) but *Gambierdiscus* cells were present. The RBA has also been useful to confirm the P-CTX-3C production by *Gambierdiscus polynesiensis* cultures and to assess variations in the toxic potency of different clones [98]. Despite the relevance of RBA as useful tool for CTX determination, the need for radiolabelled compounds has hampered the progress of these assays. Attempts have been performed to label PbTXs with other moieties such as a chemiluminescent acridinium dye [167] or different fluorescent ligands [168], resulting in promising approaches to avoid constraints associated with radioactivity.

Table 3 summarizes the biochemical assays and cell-based assays that have been developed for the detection of CTX.

**Table 3:** Biochemical assays and cell-based assays for the detection of CTX.

Assay	Detection Technique	Sample	Reference(s)
Immunoassays	Colorimetry	Fish	[124,134,135]
		-	[133,136,137,138,139,140]
	Radioactivity	Fish	[123]
Immunostick test	Colorimetry	Fish	[125,126,127,131,132]
CBA	Colorimetry	-	[142]
		Fish	[54,128,134,141,148,156,161,162,169,170]
		*Gambierdiscus* sp.	[54,76,163]
		Clams sea urchins and cyanobacteria	[54]
		Rat/mouse blood and urine	[157,158,159,160]
		Cyanobacteria	[171]
	Luminescence	-	[143]
	Fluorescence	fish	[150]
Hemolytic assay	Colorimetry	*Prorocentrum* sp. and fish	[164]
		*Gambierdiscus* sp.	[165]
RBA	Radioactivity	-	[103]
		Fish	[148,149,155,166]
		*Gambierdiscus* sp.	[155][98]
	Fluorescence	-	[168]
	Chemiluminiscence	*Gambierdiscus* sp.	[167]

### 2.3. Cyclic Imines

Cyclic imines (CIs) are a heterogeneous group of marine lipophilic phycotoxins including: spirolides (SPXs), gymnodimines (GYMs), pinnatoxins (PnTXs), pteriatoxins (PtTXs), prorocentrolides and spiro-prorocentrimines [172,173]. They are macrocyclic compounds with imine (carbon-nitrogen double bond) and spiro-linked ether moieties. They have been grouped together because of their common imine group as a part of a cyclic ring, which confers the pharmacological and toxicological activity, and due to their similar acute “fast acting toxicity” in mice [1]. CIs are produced by different dinoflagellates to a different extent: SPXs are mainly produced by *Alexandrium ostenfeldii* also known as *A. peruvianum* [174,175], GYMs are produced by *Karenia selliformis*, also known as *Gymnodinium selliforme* [176]. The PnTXs-producing organism has been described as peridinoid dinoflagellate [177] and recently identified as *Vulcanodinium rugosum* [178], prorocentrolides have been isolated from *Prorocentrum lima* [179], spiro-prorocentrimines are suggested to be produced by *Prorocentrum* species [180] and PtTXs have only been detected in shellfish and no producing organism has been identified [1].

Nowadays, the growing CIs family found in dinoflagellates and/or in shellfish includes 31 members: 4 GYMs [181,182], 14 SPXs (among which, E and F are non-toxic due to the lack of imine ring) [183], 7 PnTXs [184], 3 PtTXs, 2 prorocentrolides and 1 spiro-prorocentrimine [183,185]. Apart from these analogues, some fatty acid acyl esters derivatives, products of shellfish metabolism, have been identified: 21 SPXs [186] and 26 PnTXs [187]. Among CIs, SPXs are the largest group and together with GYMs are the best characterized. PnTXs and PtTXs are almost structurally identical and they are the CIs most closely related to SPXs. Moreover, PtTXs, prorocentrolides and spiro-prorocentrimine have been recently identified as biotransformation products of PnTxs in shellfish [184].

The first evidences indicating the presence of SPXs, GYMs and PnTxs were reported in the early 1990s during routine monitoring of bivalve molluscs in Canada [188], New Zealand [189] and Japan [190], respectively. The cyclic imine group in the molecule is assumed to be responsible for the neurotoxicity of these toxins. The mechanism of action of SPXs and GYMs is based on their inhibition of both muscarinic and nicotinic acetylcholine receptors (mAChRs and nAChRs) in the central and peripheral nervous system [36,191]. Nevertheless, while GYMs effect on these receptors is reversible, SPXs binding seems to be irreversible [185]. As regards to PnTXs, it has recently been demonstrated that they also bind to nAChRs [184]. Prorocentrolides and spiro-prorocentrimine molecular targets are still unknown [192].

Only SPXs, GYMs and PnTXs have been detected in Europe. In addition, there is limited information about the absorption, distribution, metabolism and excretion of CIs in animals or humans, and no intoxication cases have ever been reported [1]. This fact explains the absence of regulatory limits and official analysis methods for CIs in shellfish. However, the working group of the EU-RLMB has proposed a guidance level for the sum of SPXs of 400 µg/kg in shellfish meat [44,193].

Given the novelty of the research area of CIs, it is crucial to better describe their mechanisms of action, as well as to widen the toxicological and pharmacological data in order to determine if they pose a public health risk. This is particularly important considering the widespread distribution of CIs in seafood and their potent binding to nicotinic acetylcholine receptors in the central and peripheral nervous systems and this might have long-term effects on human health. It would also be important to develop suitable methodologies for the detection of all CIs and implement preventive measures in monitoring programmes [192].

#### 2.3.1. Cell-Based Assays for Cyclic Imines

Even though no CBAs have been developed so far for CIs, there are some studies about mechanism of action, which will set the basis to develop those functional tests in a near future. One of the first evidences that GYM targets muscular and neuronal nAChRs was demonstrated by Kharrat *et al.* [194]. In this work, frog and mouse nerve muscle preparations were used for electrophysiological studies by means of axonal clamp and tension experiments. GYM-A blocked the twitch response when nerve was stimulated, suggesting that this toxin should be a nAChRs blocker in muscles. Compared to tubocurarine, a 135-fold higher concentration was needed of this toxin to obtain a similar degree of GYM-A blocking. Furthermore, this blocking was concentration- and time-dependent. In order to confirm these findings in native receptors, primary cell cultures of *Xenopus laevi* embrios myocytes were used in patch clamp experiments, as these myocytes express nAChRs. Extracellular perfusion of GYM-A reduced nicotinic currents elicited by the neurotransmitter in a reversible manner, thus confirming the results obtained in nerve muscles. In addition, studies on how GYM-A affected receptors and subunits affinity were performed. Homomeric neuronalhuman α-7 nAChR were expressed in *Xenopus laevis* oocytes for electrophysiological studies (patch clamp). Moreover, neuromuscular human heteropentameric and neuronal human chimeric receptors of α-7 nAChR were expressed in HEK293 cells for competition studies. These studies revealed that GYM-A is more effective on chimeric nAChRs neuronal alfa7-5ht3 than on muscular ones, but it was 30 times less effective on homomeric α-7 nAChR expressed in *Xenopus* oocytes. The inhibitory of GYM-A was similar on different animal tissues but dependent on the nAChR receptor subtype and subunits comprising the receptor.

Dragunow *et al.* [195] studied the effects of synthetic GYM, GYM acetate and GYM carbonate on N2a cells. The toxic effect on the viability, although significant compared to the control, was very low. Nevertheless, the authors observed that pre-exposure of cells to these toxins sensitised them to okadaic acid, suggesting that GYM and its analogues may be rendering cells more susceptible to the effects caused by other toxins. However, GYM acetate did not produce stress proteins such as c-Junk, ATF-2 or ATF-3 neither proliferation, thus other mechanisms may be involved in the sensitisation of these toxins.

Another interesting study was performed by Geiger *et al.* [196], which used several cytotoxicity assays in order to investigate the mechanisms of action of PnTx-G. No viability inhibition was found on N2a, KB and CaCo2 cells even after 72 h of exposure of the toxin. Nevertheless, fractions obtained from *V. rugosum* extracts were found to be toxic for these cell lines, suggesting that other compoundscan be implied in the toxicity of *V. rugosum*.

Finally, Hellyer *et al.* [197] have recently synthesized a fluorescent PnTx-F and have applied to muscle sections from thy1-YFP-H transgenic mice, which express yellow fluorescent protein (YFP) in motor nerves, to allow visualization of interactions of this toxin with nicotinic receptors. The addition of spacers and the fluorescent moiety to PnTx-F showed reduced *in vitro* neuromuscular-blocking potency and *in vivo* toxicity compared to unmodified PnTx-F. However, despite this reduced potency, the fluorescent toxin efficaciously labelled endplates of mouse muscle motor nerves, retained neuromuscular blocking abilities *in vitro*, and displayed *in vivo* toxicity. Consequently, this study is an important step not only to provide with fluorescence assays for toxin detection, but also to understand how toxins and derivatives interact with nicotinic receptors.

Tatters and co-workers [198] performed a hemolytic study based on Eschbach erythrocyte lysis assay [199] for ichthyotoxic algae, and quantified the response in relation to saponin. A weak hemolytic activity was observed for the GYM standard, whereas *Karenia selliformes* reported a high response. Hemolytic assay appeared to be a non-sensitive method for GYM detection.

#### 2.3.2. Receptor-Binding Assays for Cyclic Imines

The birth of biochemical methods for the detection of CIs is very recent. The first work was a FP indirect assay in solution for the detection of GYM-A and 13-desmethyl C (13-DesMeC) SPX [200]. The authors developed a competitive inhibition assay based on the affinity of these toxins towards nAChRs, purified from *Torpedo marmorata* electrocyte membranes, and using fluorescent α-bungarotoxin (BTX) as a competitive tracer. The assay provided IC50 values of 390 nM and 108 nM for GYM-A and 13-DesMeC SPX, respectively. Matrix effects were evaluated using mussel samples, which only shifted slightly the IC50 values to 281 and 129 nM, respectively. The higher sensitivity, and thus affinity, of SPX towards the receptors compared to GYM, suggests an also higher toxicity. The same authors also demonstrated the suitability of this assay to the quantification of 13,19-didesmethyl C (13,19-diDesMeC) SPX, with a sensitivity similar to that of 13-DesMeC SPX, and negligible matrix effects [201]. The applicability of this assay to clams, cockles and scallops has also been demonstrated [202]. Spiked shellfish extracts provided IC50 values of around 100 nM for SPXs and between 300 and 400 nM for GYMs. Otero *et al.* [203] labelled nAChRs with a derivative of fluorescein to develop a FP direct assay for the quantification of SPX. The approach provided appropriate LODs for 13-DesMeC SPX and 13,19-diDesMeC SPX (25 and 150 nM, respectively). The shift from indirect to direct recognition implied a significant improvement of sensitivity and rapidity.

Based on the same sensing principle, solid-phase RBAs with different detection techniques have been developed [204,205,206,207]. Rodríguez and co-workers [204] developed an indirect assay based on a competition between biotin-labelled α-BTX and CIs for their binding to nAChRs in solution, and the subsequent immobilisation of the α-BTX-receptor complex on streptavidin-coated surfaces. The amount of immobilised receptor was measured by using an anti-nAChR Ab and an HRP-labelled secondary Ab. The use of three different substrates for HRP allowed adapting the assay to chemiluminescence, fluorescence and colorimetric detection techniques. The three end-points yielded similar calibration curves for 13-DesMeC SPX (IC50 ≈ 35 nM and LOD (IC20) ≈ 10 nM), although the chemiluminescence substrate seem to be slightly better. Moreover, no matrix interferences were observed when using cockle extracts. The chemiluminescence approach was also applied to the detection of GYM, being 10 times less sensitive than with 13-DesMeC, possibly due to the lower GYM toxicity. The same authors developed a similar assay, but first immobilising the biotin-labelled α-BTX on streptavidin-coated plates and then carrying out the competition step and the primary and secondary Ab incubations [205]. The assay was successfully applied to the determination of 13-DesMeC SPX, 13,19-diDesMeC and 20-MetG (IC50 = 38, 18 and 55 nM and LOD (IC10) = 11, 8 and 16 nM, respectively), the first one also tested in scallops. Additionally, the assay was optimized in 384-well plates for high-throughput screening.

The immobilisation of the receptors instead of the α-BTX has also been exploited for the development of two assays [206,207]. In the first work, the competition takes place between biotin-labelled α-BTX and 13,19-diDesMeC, 13-desMeC, PnTX-G, PnTX-A, GYM-A or AK-PnTX-A for binding to nAChRs immobilised on microtiter plates, and the colorimetric detection is measured using streptavidin-HRP [206]. In order to address the lack of selectivity of the assay, the toxins bound to nAChRs were eluted from the wells and analyzed by mass spectrometry to determine their chemical structure. In the second work, nACHRsor AChBPs (binding proteins) were immobilised on carboxylated microspheres and the subsequent binding competition between biotin-labelled α-BTX and 13-DesMeC was measured by flow cytometry using fluorescent phycoerythrin (PE)-labelled streptavidin [207]. Both receptors allowed the detection of 13-desMeC SPX, 13,19-DesMeC SPX and 20-MetG SPX and GYM with different sensitivities but always in the nanomolar range, attaining even an LOD as low as 0.05 nM for 13-desMeC SPX in scallops.

Table 4 summarizes the biochemical assays and cell-based assays that have been developed for the detection of CIs.

**Table 4:** Biochemical assays and cell-based assays for the detection of CIs.

Assay	Detection Technique	Sample	Reference(s)
CBA	Colorimetry	-	[195]
		*Vulcanodinium rugosum*	[196]
	Patch clamp (electrophisiology)	-	[194]
Hemolytic assay	Colorimetry	*Karenia selliformis*	[198]
RBA	Colorimetry	Cockles	[204]
		Clams, oysters, scallops and mussels	[206]
	Fluorescence	Cockles	[204]
	Fluorescence (coupled to flow cytometry)	Scallops	[207]
	FP	Mussels	[200,201,203]
		Clams, cockles and scallops	[202]
	Chemiluminiscence	Cockle	[204]
		Scallops	[205]

### 2.4. Tetrodotoxins

Tetrodotoxin (TTX) is one of the most potent low-molecular-weight marine neurotoxins (319 Da). Its chemical structure was described as a cage-like polar molecule with a cyclic guanidinium moiety fused to a dioxy-adamantane skeleton embellished by six hydroxyl groups. To date, 29 different analogues of TTX have been reported. Their degree of toxicity varies among analogues, although not much is known about them [208]. An important recognized feature is that the deoxy analogues of TTX are less toxic than TTX, while the hydroxyl analogues are more toxic than TTX [209].

TTX was first discovered in 1909 by Tahara in ovaries of globefish [210] and its structure was more understood in 1950 with the isolation of the crystalline form of TTX from the ovaries of puffer fish (*Fugu rubripes*) [211]. However, the complete structure was only known through the findings of three independent groups [212,213,214]. Puffer fish seems to accumulate the toxin in the gonads, liver and skin [215]*.* Later on, the presence of TTX has been reported in many invertebrate species, such as starfish, blue-ring octopus, xanthid crabs, gastropod and flatworm, and in vertebrate species, including frogs, goby and newt Taricha [216]*.* TTX is produced by certain endo-symbiotic bacteria, such as *Vibrio* sp., *Pseudomonas* sp. and *Alteromonas* [217].

Like saxitoxin (STX), TTX has the ability to selectively bind to VGSCs, blocking both nerve and muscle action potentials. Contrary to CTX, TTX closes the channels. This blocking affects the passive influx of sodium ions, resulting in numbness, respiratory paralysis, mild gastrointestinal effects, and even death of human consumers. Consequently, bioaccumulation of TTX in seafood and subsequent entrance in the human food chain poses a real risk to human safety.

The Japanese government has established a regulatory limit of 2 mg/kg of TTX equivalents in food, due to “fugu” consumption in Japan. In contrast, no regulatory limits have been set in Europe because TTX poisoning had not been a problem. However, in October 2007, the first toxic European episode were reported in Malaga (Spain), caused by the ingestion of a trumpet shell of the species *Charonia lampas lampas.* The product was purchased in a Malaga market, but it was caught in the south coast of Portugal [218]. Again, in 2005 and 2008, puffer fish migrating from the Red Sea to the Mediterranian Sea due to the Lessepsian phenomenon, were responsible for human poisoning, although low levels of TTX were found in liver and gonades [219]. Other seafood species such as molluscs and echinoderms were caught in the Portugal coast and have also been described to contain TTX [220]. Therefore, European seafood is endangered of being contaminated with this hazard toxin, which highlights the need to have a regulation and to develop unambiguous, fast and reliable methods to specifically detect and quantify TTX in order to protect human health.

#### 2.4.1. Immunoassays and Immunosensors for Tetrodotoxins

Like with CIs, in the development of immunoassays for TTX, production of Ab is a bottleneck because of the small size of the toxin and the need to conjugate it to a carrier protein in order to get animal immunisation. Ideally, the carrier protein to be used as a coating agent in the ELISA should be different from that used for animal immunisation in order to avoid cross-reactivity of the Ab against the protein [221]. As an example, TTX-BSA conjugate has been synthesized for MAb production and TTX-OVA conjugate has been used as a coating agent in ELISA [221], and vice versa [222,223]. The developed competitive ELISAs have attained proper LODs, e.g., 2 [221,223,224], 5 [225] and 10 ng/mL [226], regardless the experimental conditions and formats.

Another important feature in immunoassays is the cross-reactivity towards analogues. Not much work has been performed in this direction, due to the fact that standards are not commercially available. Although some TTX derivatives may be chemically synthesized or extracted and purified from naturally-contaminated material, the cross-reactivity values may not be accurate. Nevertheless, useful qualitative information has been provided. Kawatsu and co-workers [221] reported no cross-reactivity of their Ab towards gonyautoxins or tetrodonic acid, and minor cross-reactivity towards anhydro-tetrodotoxin. In fact, the cross-reactivity of an Ab depends on the functional group used as a site to conjugate the toxin to the carrier protein. The observed results are consistent with the fact that the guanidinium group was used to conjugate TTX to BSA.

Some direct ELISA formats have been developed in order to reduce analysis times. Neagu and co-workers [224] produced a TTX-ALP tracer to use in an ELISA with immobilised anti-TTX MAb. The Ab was still able to recognize the tracer, indicating that the conjugation did not affect much the affinity event. Nevertheless, it is necessary to mention that, even though the detection was direct, a secondary Ab (non-labelled) was used to improve the anti-TTX MAb orientation and, therefore, the analysis time was still compromised. Other works have conjugated HRP [221,227] or ALP [223,228] to the anti-TTX antibody to avoid the use of a labelled secondary Ab. Despite the advantages of using conjugates, their stability (of the linkage and of the enzyme activity) still is a pending task. Recombinant Ab fragments have also been synthesized through phage display technology, and used in the development of ELISAs for TTX determination [227]. However, in order to achieve high sensitivities, more work needs to be undertaken.

Some of these direct ELISA configurations have been used in the development of electrochemical immunosensors on screen-printed electrodes. In the approach with the TTX-ALP tracer [224], differential pulse voltammetry (DPV) was used to measure the *α*-naphthol produced by the reaction between the substrate α-naphthyl phosphate and the enzyme label, decreasing the LOD from 2 to 1 ng/mL (compared to the colorimetric approach). In the approach with labelled primary Ab [228], based on the amperometric detection of the *p*-aminophenol at +300 mV *vs.* Ag/AgCl, product of the reaction between *p*-aminophenyl phosphate and ALP, an LOD of 0.016 ng/mL was attained, one of the lowest limits ever reported.

Immunochromatographic strips have also been developed with the aim of providing a rapid visual test for the screening of TTX [229,230,231]. These assays, prepared on an absorbent pad to favor lateral flow, are based on the competition between the TTX from the sample and the TTX-BSA immobilized on the test line for a colloidal gold-labelled anti-TTX Ab. The visual LOD attained with this format using spiked puffer fish samples (muscle and gonad) was 40 ng/mL of TTX [229,230]. Although this method is not quantitative, it has the advantages of simplicity, ease of use, no sophisticated equipment requirement and short analysis times (10 min). This test has also been used in the screening of fish, establishing an action limit of 2 mg/kg [231]. The comparison with LC-MS/MS analysis has demonstrated the applicability of strip tests to the analysis of naturally-contaminated fish samples.

Several SPR immunosensors for the detection of TTX based on direct approaches have been reported [232,233,234,235,236,237]. Due to the small size of the toxin, it is very difficult to directly detect its interaction with immobilised Ab and, consequently, the appropriate configuration involves antigen immobilisation and detection of the Ab interaction. In the first works [232,233,234,235], the gold SPR chip was functionalised with a mixed self-assembled monolayer (SAM) of hydroxy- and amino-terminated oligo-ethylene glycol alkanethiols (OEG-ATs), since the ethylene glycol units were known to minimize non-specific protein adoption. Whilst amino-terminated OEG-ATs were used to covalently link the TTX to the surface with formaldehyde, hydroxy-OEG-ATs were used as spacer molecules to avoid cross-linking between amino-OEG-ATs. TTX was also linked using reductive alkylation but, as expected, higher Ab recognition yields were observed when using formaldehyde because the reaction site (the guanidine group) was the same as in the Ab production. TTX has also been immobilised directly on a carboxymethylated chip, which simplifies the protocol and still retains the performance [236]. Like in colorimetric competition immunoassays, the MAb concentration was optimized taking into account that surface saturation is not desired, since low TTX concentrations would be more difficult to detect. These optical immunosensors attained a LOD (IC20) as low as 0.3 ng/mL [233]. It is evident that the most exhaustive applicability studies have been performed with SPR immunosensors. The analysis of matrix effects from pufferfish liver and muscle, gastropods, and urine, is of utmost importance in food safety and clinical applications. Although the presence of a natural matrix may increase the LOD, this effect can be minimized by properly modifying the assay running buffer [234]. Several experimental parameters, such as pH, concentration and BSA presence, can be modulated to avoid non-specific adsorption while maintaining an appropriate Ab interaction and LOD. The analysis of naturally-contaminated fish has always shown appropriate correlations with LC/MS analysis [51,234]. An additional advantage of these systems is the ability to regenerate the surface for continuous TTX determinations. A pre-validation study was performed in parallel by three independent laboratories using spiked and naturally-contaminated pufferfish samples [235]. Although the method could be improved and the comparison was only a proof of concept, it is reasonable to conclude that SPR immunosensors are very promising as analysis tools for TTX quantification.

The SAM immobilisation strategy has also been used in the development of another optical immunoassay for TTX [238]. In this case, the chip was inserted into a microfluidic cell coupled to a microscope and a camera, and the interaction was monitored by imaging using a microbead-coated secondary Ab. Controlled fluidic forces were applied to remove non-specifically bound beads by fluidic force discrimination (FFD). The LOD for TTX was 15 ng/mL and a large dynamic range (4–5 orders of magnitude) was obtained.

Recently, Yakes and co-workers [237] have developed a non-competitive SPR immunosensor, immobilising anti-TTX MAb on the surface of the SPR chip instead of TTX. This new configuration permits the direct detection of free TTX, which is a challenge taking into account its low molecular weight. This has been possible thanks to the recent advances in SPR instrumentation: lower noise detection systems, improved fluidics with stronger vacuum pumps and lower noise valves. Antibody kinetics and cross-reactivity studies towards other co-occurring toxins with a similar mode of action were performed, which demonstrated the selectivity of the antibody, since no competitive or additive effects were observed. This strategy has allowed lowering the LOD with respect to the competitive SPR biosensors to 0.09 ng/mL of TTX and could, in a future, be extended to the detection of other small molecules.

#### 2.4.2. Cell-Based Assays and Biosensors for Tetrodotoxins

The first N2a CBA for TTX (and also STX) detection was developed by Kogure and co-workers [239], and was initially named tissue culture-based assay (TCBA) because of the cell tissue layer created on the well. Previous studies had suggested that TTX blocked conduction of nerve and muscle through selective inhibition of the sodium-carrying mechanism [240,241]. The assay was thus based on the ability of TTX to block VGSCs and rescue cells from the effect of ouabain (which inhibits the Na^+^/K^+^-ATPase pump) and veratridine (which causes sodium ion influx into the cells), counteracting the toxic antagonist effect of veratridine and increasing the viability of cells. In that assay, viability was measured by the morphological changes of the cells with an optical microscope, attaining an LOD of 3 nM of TTX. The assay was applied to the analysis of strains of bacteria from marine sediments. The applicability of the assay to freshwater sediments was also demonstrated [242], indicating that TTX can be produced by freshwater bacteria and accumulate in lakes and ponds sediments. The assay has also been useful to screen the TTX production by bacteria isolated from the ovaries of pufferfish [243].

In order to get more accurate quantifications, the assay has been improved by specific staining of the cells with dyes, which estimate the relative abundance of living cells by absorbance measurements. With this aim, neutral red [244] and tetrazolium salts such as WST-1 [245] and MTT [246] have been used, these salts avoiding the washing steps thus providing greater reproducibility. The IC50 values attained with these dyes were 50, 6.6 and 12.9 nM, respectively.

When investigating the applicability of the CBA for TTX; it is necessary to keep in mind that this assay is not specific for TTX but can also detect other VGSC blockers such as STX and gonyautoxins; which could co-occur with TTX in the same sample [247,248,249]. Nevertheless; this CBA has demonstrated to be useful as a screening method. In addition, of interest is the application of CBAs to assess the toxicity of TTX analogues [250].

Following the same principle but a different measurement technique, a biosensor for TTX detection has been developed [251]. This biosensor consisted of a sodium electrode covered with frog bladder membrane integrated within a flow cell. The concentration of TTX was measured from the inhibition ratio of the sensor peak output. When naturally-contaminated puffer fish samples were analyzed, linear correlations were obtained in the comparison with MBA, the biosensor being able to detect TTX at concentrations far below the LOD of the MBA (1 µg/g). Again, the biosensor was also able to detect STX and GTX analogues [251].

In a more sophisticated work, a portable microelectrode array incorporating neuronal networks has been developed for TTX detection [252,253]. The system was able to monitor extracellular potentials from spinal cord cells cultured on electrodes and exposed to TTX, attaining an IC50 of 4 nM of TTX. Similar is the work performed by Mohan and co-workers [254], who used patch clamp electrophysiology to record the action potentials caused by the effect of TTX on NG108-15 cells. In this case, experimental data were used to create a computer model. Despite the fact that the toxic effect of TTX was observed, more efforts are needed to provide a proper calibration curve and to refine the mathematical model. Comparison between electrophysiological recordings and a CBA with a fluorescent dye and has been performed [255], using recombinant subunits expressed by Chinese hamster ovary (CHO) cells. There was a linear relationship between the log IC50 values obtained by the two methods for different VGSC modulators.

Like for CTX, the hemolytic capacity of TTX has been exploited for the development of a screening assay [164], based on the N2a CBA for CTX [141] and the heamolytic assay for PLTX [70]. In this test veratridine was used as TTX antagonist. The hemolytic assay was performed with red tilapia RCBs. This fish has the capacity to regulate his body ion concentration to a wide range of salinities. More experimentation is required to find minimal concentration of TTX needed to inhibit hemolysis reactive concentrations for a valid detection. Table 5 summarizes the biosensors, biochemical assays and cell-based assays that have been developed for the detection of TTX.

**Table 5:** Biosensors, biochemical assays and cell-based assays for the detection of TTX.

Assay/Biosensor	Detection Technique	Sample	Reference(s)
Immunoassay	Colorimetry	-	[222,224,226,227]
		Puffer fish	[221,223,225]
Immunosensor	Electrochemistry	-	[224,228]
	SPR	-	[233,237]
		Pufferfish	[232,234,235]
		Sea snail	[236]
		Human urine	[234]
		Milk and apple juice	[232]
	FFD	-	[238]
Immunostick test	Visual	Spiked puffer fish	[229,230]
		Fish	[231]
CBA	Colorimetry	-	[239]
		Bacteria from freshwater sediments	[242]
		Bacteria from pufferfish	[243]
		Bacterial culture supernatants	[244,245]
		Spiked *E. coli*	[246]
		Newts	[250]
	Fluorescence	-	[255]
	Patch clamp (Electrophysiology)	-	[252,253,254,255]
		Pufferfish	[251]

## 3. Conclusions and Future Perspectives

When available, the instrumental analysis approach for the quantification of emerging toxins is suitable for the unambiguous quantification of analytes. However, if the potency of each analogue has not been described (*i.e.*, toxicity factors between analogues are not well established), the instrumental analysis methods may not be the right solution to estimate toxicity and risk. As MBAs are controversial, alternative methods providing functional or toxicological information have a role to play in the identification of risks, as a complement to instrumental analysis approaches. Alternative methods based on structural recognition can also contribute to quantify overall presence of analogues of a group of toxins. Therefore, alternative methods, once validated, may become excellent screening tools and even contribute to quantify toxins or restricted groups of toxins. Instrumental analysis methods may be applied to obtain more precise quantifications or toxin profiles in positive samples identified by screening assays.

Whereas some alternative methods for the detection of classical marine toxins have even reached the market (e.g., protein phosphatase inhibition assay (PPIA) for okadaic acid and ELISA for domoic acid), those for emerging marine toxins, although successful, have not been optimized nor validated, and those few that have reached the market, such as the immunostrip tests for CTXs, have suffered from inefficiencies and controversy. A reason for this may be that not enough is known about the toxicity and mechanism of action of some emerging toxins, this being especially important when considering families of toxins with numerous analogues. This scarce information, together with the lack of certified standards and reference materials, are responsible for the underdevelopment of analysis methods. As a consequence, official methods and MPLs of these toxins in seafood may not have been established yet. Additionally, for some of these toxins, toxicological evidence is not available yet and thus, regulatory institutions cannot adopt appropriate regulations.

Each alternative method has advantages and drawbacks. The interaction of toxins with antibodies is based on a structural recognition, thus not necessarily related to toxicity. However, antibodies are still robust biorecognition molecules, with high affinity and sensitivity towards their analytes, sometimes being even able to detect different analogues of the same group of toxins. Moreover, their easy handling and manipulation allow their integration into different assay formats, being adaptable to the different end users. Like antibodies, receptors specifically bind to their corresponding ligands on a structural recognition basis. The replacement of the commonly used radioactive labels by optical ones has favored the spreading of RBAs. However, they still suffer from complex setups and the requirement of the isolation of receptors from a variety of animals. Cells provide an indication of the overall toxicity, and have the advantage to be closer to animal models than antibodies and receptors. The use of human cell models can be even more useful to extrapolate some effects on human tissues. However, working with “live” material implies certain degree of variability, both inter and intra cell lines. Environmental conditions, cell status or cell-cycle stage can also lead to variations in the responses. Another limitation is the impossibility or difficulty to identify or discriminate compounds that share the same mechanism of action. The effects from the seafood matrices on the assay or biosensor are also of concern in any detection method.

It is evident that the alternative methods described in this review are promising tools to identify and quantify emerging marine toxins. Some of them have even been demonstrated to be highly performing and applicable to the analysis of naturally-contaminated samples, and when limitations have been identified, efforts have been made to overcome them. As an example, sample fractioning can separate toxins according to their chromatographic retention time and thus, contribute to discriminate them. As another example, matrix effects can be minimized by modifying toxin extraction protocols or adding previous sample clean-up steps. However, attempts to optimize, validate and standardise these methods have been scarce, so far. Now, it is about time to make efforts to solve the pending tasks. The combination of screening tools and confirmatory instrumental analysis methods should be the solution to achieve highly specific, sensitive and fast routine monitoring of emerging marine toxins. Additionally, research has to pursue to better describe mechanisms of action of emerging toxins, to identify or produce further recognition molecules, and to develop sample purification methods in order to promote the implementation of alternative methods. It is also necessary to take into account that these alternative methods will be used by laboratories performing environmental and food safety controls, food producers, fishermen and other end-users. Consequently, their formats should be friendly and easy to use. The development of kits is certainly a sound and reachable goal, as it has happened with some regulated toxins (PSP, ASP and some lipophilic toxins). However, for some experimental approaches such as CBAs, the development of kits may not be appropriate, and in this case the priority would be the coordination of laboratories in order to harmonise and validate the methodologies. Biosensors are a very promising approach since they have demonstrated to be very specific and robust, to attain very low limits of detection, and to conduct analyses in short times. Depending on the type and the format, they may provide additional advantages such as real-time responses and, when miniaturisation is possible, device portability for *in situ* measurements. Multi-disciplinary and cooperative work would certainly speed up and strengthen the progress in this area.
